# Trop2-targeted therapies in solid tumors: advances and future directions

**DOI:** 10.7150/thno.98178

**Published:** 2024-06-11

**Authors:** Xinlin Liu, Leina Ma, Jiyixuan Li, Li Sun, Ying Yang, Ting Liu, Dongming Xing, Saisai Yan, Miao Zhang

**Affiliations:** 1 The Affiliated Hospital of Qingdao University, Qingdao University, Qingdao 266071, China.; 2 Qingdao Cancer Institute, Qingdao, 266071, China.; 3 School of Life Sciences, Tsinghua University, Beijing, 100084, China.

**Keywords:** Trop2, targeted therapies, solid tumor, therapeutic efficacy

## Abstract

Trophoblast cell surface antigen 2 (Trop2) is overexpressed in a range of solid tumors and participants in multiple oncogenic signaling pathways, making it an attractive therapeutic target. In the past decade, the rapid development of various Trop2-targeted therapies, notably marked by the advent of the antibody-drug conjugate (ADC), revolutionized the outcome for patients facing Trop2-positive tumors with limited treatment opinions, such as triple-negative breast cancer (TNBC). This review provides a comprehensive summary of advances in Trop2-targeted therapies, including ADCs, antibodies, multispecific agents, immunotherapy, cancer vaccines, and small molecular inhibitors, along with in-depth discussions on their designs, mechanisms of action (MOAs), and limitations. Additionally, we emphasize the clinical research progress of these emerging Trop2-targeted agents, focusing on their clinical application and therapeutic efficacy against tumors. Furthermore, we propose directions for future research, such as enhancing our understanding of Trop2's structure and biology, exploring the best combination strategies, and tailoring precision treatment based on Trop2 testing methodologies.

## Introduction

Trophoblast cell surface antigen 2 (Trop2), identified in 1981, was a type I surface glycoprotein that has garnered attention in oncology 1. It is also known by various other names including epithelial glycoprotein-1 (EGP-1), gastrointestinal tumor-associated antigen GA7331, pancreatic carcinoma marker protein GA733-1/GA733, CAA1, membrane component chromosome 1 surface marker 1 (M1S1), and TTD2 2-4. Trop2 is critical in embryonic organ development, and its expression is limited in normal tissues 5. Accumulating evidence suggests that Trop2 is overexpressed in a range of solid tumors, significantly impacting tumor growth, invasion, and metastasis 6, 7. Consequently, Trop2 has become an attractive prognostic marker and therapeutic target for solid tumors. The approval of the first Trop2-targeted antibody-drug conjugate (ADC), Trodelvy^TM^ (sacituzumab govitecan), which enhances survival in metastatic breast cancer (mBC) and metastatic urothelial cancer (mUC), has encouraged further research on Trop2 biology and the expansion of Trop2-targeted therapeutic opinions. Despite its potential, poor response rates and unfavorable risk-benefit profiles present ongoing challenges. As a result, extensive research continues in preclinical, translational, and clinical settings to innovate and enhance targeted therapies for Trop2.

Progress in Trop2 targeting involves advancing ADC technology, modifying linkers, payloads, or antibody scaffolds to optimize therapeutic efficacy 8. Other approaches encompass the development of antibodies, multispecific agents, immunotherapies, cancer vaccines, and small molecule inhibitors. In this review, we present an unprecedented overview of Trop2-targeted therapies, with a focus on their designs, mechanisms of action (MOAs), and limitations. We also highlight the clinical advancements of novel therapies, which could alter the treatment landscape of Trop2-positive tumors. Finally, we maintain an optimistic view regarding the future of Trop2-targeted therapies, envisioning a growing emphasis on Trop2's structure and biology, combination strategies, and biomarker-driven precision treatment that will contribute to improved clinical outcomes for patients with Trop2-positive tumors.

## Trop2-targeted therapies

**Figure 1:**
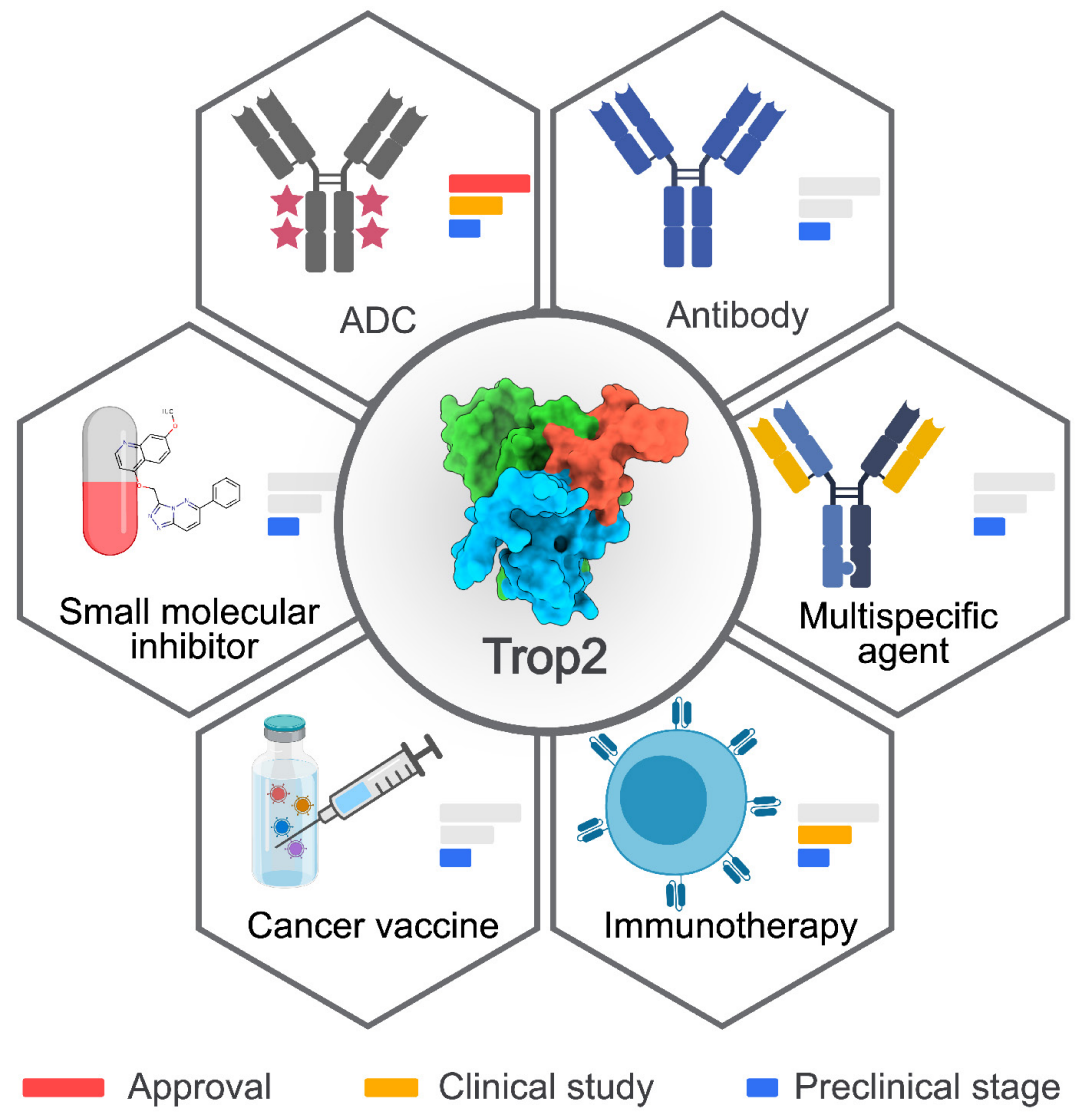
** Summary of Trop2-targeted therapies. Six types of Trop2-targeted therapeutics have been developed.** The development phases are depicted with a red bar for approval, a yellow bar for clinical studies, and a blue bar for the preclinical stage. The crystal structure of Trop2-ECD (7E5M) was obtained from Protein Data Bank (PDB). The image was created with* MedPeer.cn*.

Since the initial approval of Trodelvy^TM^ for the treatment of triple-negative breast cancer (TNBC) and mUC, a range of Trop2-directed therapies with different MOAs and safety profiles have been developed (Figure 1). Next, we summarize their therapeutic outcomes and discuss the designs, MOAs, and limitations of each strategy.

### Trop2-directed ADCs

In light of Trodelvy^TM^'s success, over fifteen Trop2-directed ADCs are currently undergoing clinical investigation with the goal of improving therapeutic efficacy. Below, we discuss in detail key examples of novel ADCs, and additional ADCs in clinical development are listed in Table 1.

**Sacituzumab govitecan (Trodelvy^TM^).** Sacituzumab govitecan (SG), developed by Immunomedics and Gilead Science, is the only approved Trop2-targeted agent. It consists of a humanized IgG1κ anti-Trop2 antibody (hRS7), a hydrolyzable CL2A linker, and the active metabolite of irinotecan (SN-38) 9, 10. The preclinical study demonstrated that SG exerts significant and specific antitumor effects against various Trop2-positive tumor types. Additionally, this first-in-class (FIC) Trop2-targeted ADC is well tolerated in monkeys at clinically relevant doses 11. Multiple unique attributes, including a high drug-to-antibody ratio (DAR) of 7.6, a moderately potent payload, and a high dosing regimen, contribute to the favorable therapeutic index of SG 12. Numerous clinical trials have shown favorable outcomes with SG, leading the approval from the Food and Drug Administration (FDA) 13-20. To date, SG has been approved for three indications, including unresectable locally advanced or metastatic TNBC, unresectable locally advanced or metastatic hormone receptor (HR)-positive, human epidermal growth factor receptor 2 (HER2)-negative breast cancer (HR^+^/HER2^-^ BC), and locally advanced or mUC.

Currently, more than 40 clinical trials are ongoing or planned to explore the efficacy of SG, alone or in combination with other therapies, across various solid tumor types, especially those with limited treatment opinions. It is heartening to note that an impressive objective response rate (ORR) of 70% was achieved in the Double Antibody Drug Conjugate (DAD) phase I trial, which was designed to evaluate the combination of SG and a nectin-4-directed ADC enfortumab vedotin (EV) in mUC. Given the promising clinical results of SG + EV, further trials have been planned to investigate triplet therapy of SG in combination with EV and pembrolizumab in treating mUC. Although SG has enhanced responses and outcomes for patients with metastatic TNBC and mUC, the prevalence of SG-related adverse events (AEs) has significantly risen between 2020 and 2023, coinciding with its expanded clinical use. The predominant AEs reported in mTNBC clinical trials involving SG treatment included diarrhea, neutropenia, leukopenia, nausea, anemia, constipation, fatigue, alopecia, and vomiting. Approximately 60% of patients treated with SG experienced diarrhea, with around 10% suffering from severe (grade 3) episodes, likely due to the drug dissociating early from its antibody, leading to off-target off-tumor toxicity 16, 20. Liu *et al.* identified an association between SG and an increased risk of blood lymphatic system disorders and hepatobiliary disorders 21. Additionally, they noted that the median onset time for SG-related AEs was 14 days, with the bulk of these events emerging within the first month of treatment, although some AEs continued to manifest up to six months later. Consequently, extended follow-up periods will be essential to monitor SG-related AEs in upcoming clinical trials. Likewise, resistance to SG continues to be a significant challenge. One reason for SG treatment failure is the lack of Trop2 expression. The T256R mutation of Trop2 can lead to decreased plasma membrane localization and reduced cell-surface binding by hRS7, resulting in acquired resistance to SG 22. In January 2024, Gilead announced that the phase 3 EVOKE-01 study did not meet its primary endpoint of overall survival (OS) in previously treated metastatic non-small cell lung cancer (NSCLC). In May 2024, the TROPiCS-04 phase 3 confirmatory study focusing on locally advanced or mUC failed to meet the primary endpoint of OS in the intention-to-treat population. The experience with NSCLC and mUC indicates that replicating the success seen in breast cancer treatment is challenging due to differences in tumor biology and Trop2 expression levels.

**Datopotamab deruxtecan (Dato-DXd).** Dato-DXd, a novel Trop2-directed ADC, has been developed by Daiichi Sankyo and AstraZeneca. It consists of a recombinant humanized anti-Trop2 IgG1 monoclonal antibody (mAb) conjugated with a topoisomerase I inhibitor (DXd) via a tetrapeptide-based linker, which attaches to reduce cysteine residues at the interchain disulfide bonds of datopotamab 23. High internalization activity, DNA damage, and apoptosis induced by Dato-DXd were observed in Trop2-expressing tumor cells *in vitro*. Dato-DXd further showed potent antitumor efficacy in a broad range of xenograft tumors *in vivo*, including patient-derived xenograft (PDX) models of NSCLC. Additionally, Dato-DXd exhibited well tolerability in rat and monkey models. Unlike other ADCs, Dato-DXd was designed with an optimized DAR of 4, which helps to maximize the therapeutic window in preclinical studies. To date, 18 clinical trials have been initiated to evaluate the therapeutic efficacy and safety of Dato-DXd in various solid tumors, including TNBC, HR-positive, HER2-low or -negative breast cancer (BC), and NSCLC. The primary results of a first-in-human (FIH) phase 1 clinical study (ClinicalTrials.gov identifier: NCT03401385) revealed that Dato-DXd showed promising antitumor activity and a manageable safety profile in heavily pretreated patients with advanced NSCLC 24. The ORR was 26%, with an OS of 6.9 months. The TROPION-Lung01 phase 3 study (NCT04656652) indicated a significant improvement in progression-free survival (PFS) with Dato-DXd compared to docetaxel (DTX) in previously treated advanced/metastatic NSCLC 25. Additionally, the phase 1b clinical study TROPION-Lung02 (NCT04526691) demonstrated that the combination of Dato-DXd and pembrolizumab exerted promising first-line (1L) activity with an ORR of 60% in advanced NSCLC 26. Initial results from the phase 2 TROPION-Lung05 trial (NCT04484142) showed that Dato-DXd exhibited encouraging antitumor activity with a confirmed ORR (cORR) of 35.8% in heavily pretreated NSCLC patients with actionable genomic alterations (AGAs) 27. Overall, Dato-DXd has demonstrated tremendous potential and a manageable safety profile in the treatment of various Trop2-positive solid tumors, with particular promise shown in NSCLC. While the clinical potential of Dato-DXd is promising, its side effects require diligent attention and management. Interstitial lung disease (ILD) is a significant identified risk associated with Dato-DXd, especially in patients with NSCLC. During the TROPION-PanTumor01 clinical trial, 11% of patients experienced drug-related ILD 28. Careful monitoring and prompt intervention are necessary for ILDs to minimize the risk of serious health outcomes. Prophylactic measures are essential to prevent infusion-related reactions (IRRs), oral mucositis, stomatitis, ocular surface events (OSEs), and nausea and vomiting.

**SKB264.** SKB264 is a Trop2-targeted ADC that is developed by Kelun-Biotech. Sharing the same mAb as SG, SKB264 uses a sulfonyl pyrimidine-CL2A-carbonate linker to conjugate the mAb with seven to eight molecules of a payload named KL610023, a topoisomerase I inhibitor derived from Belotecan. Like other ADCs, SKB264 is engineered to release a free payload that inhibits tumor cell proliferation following internalization prompted by Trop2-specific binding 29. Preclinical data showed that SKB264 exhibited potent inhibitory activity with a nanomolar level of inhibitory concentration IC_50_ in various Trop2-positive tumor cell lines. Furthermore, SKB264 significantly inhibited tumor growth in a dose-dependent manner in both cell-derived xenograft (CDX) and PDX models *in vivo*. The pharmacokinetic-pharmacodynamic (PK-PD) study demonstrated that SKB264, with its longer half-life, had better antitumor efficacy than SG. This data suggests that SKB264 has achieved a favorable balance between stability in circulation and release of the payload within tumor cells. Building on these positive outcomes, eight clinical trials are underway or planned to assess the therapeutic effect of SKB264, alone or in combination with other agents, in multiple advanced solid tumors, such as NSCLC, TNBC, and HR^+^/HER2^-^ BC. Recently, the primary results from the clinical trial of SKB264 in advanced NSCLC patients (NCT04152499) were presented at the 2023 ASCO conference 30. In the study, the ORR of 44% and median duration of response (DoR) of 9.3 months were observed among the 39 response-valuable patients. Notably, in the subgroup with tyrosine kinase inhibitor (TKI) -resistant epidermal growth factor receptor (EGFR) -mutant NSCLC, an ORR of 60% and a median PFS of 11.1 months were recorded. Additionally, there were no reported cases of neuropathy or drug-related ILD/pneumonitis. The phase 2 clinical study of SKB264 in patients with locally advanced or metastatic TNBC also showed encouraging antitumor activity, demonstrated by an ORR of 42.4% and a manageable safety profile. Results from the phase 3 OptiTROP-Breast01 study (NCT05347134) in patients with advanced TNBC showed that SKB264 monotherapy significantly improves PFS and ORR compared to chemotherapy. Additionally, it maintains a manageable safety profile in patients with heavily pretreated TNBC and few treatment alternatives. Kelun-Biotech has submitted a listing application of SKB264 in China for the treatment of adult patients with unresectable locally advanced or metastatic TNBC who have received at least two prior systemic therapies, including at least one for advanced or metastatic disease.

**PF-06664178 (RN927C).** PF-06664178, a site-specific Trop2-targeted ADC with enhanced stability, was first reported by Pfizer in 2016 31. This ADC is composed of a humanized anti-Trop2 IgG1 antibody (RN926) conjugated to an AcLys-VCAur0101 linker-payload. Aur0101 is a potent tubulin polymerization inhibitor with an IC_50_ in the picomolar range. In consideration of the balance of efficacy and toxicity, a low DAR of 2 was chosen for PF-06664178. Preclinical studies demonstrated robust *in vitro* cytotoxicity of PF-06664178 against Trop2-positive tumor cell lines and promising *in vivo* antitumor efficacy in a range of PDX models, including TNBC, ovarian, and lung cancers 31. Toxicity signals, it should be noted, were observed in Trop2-expressing normal tissues of cynomolgus monkeys. Increased necrosis was observed in various epithelial tissues, including skin, upper gastrointestinal (GI) mucosa, and vaginal tissues. In 2014, a phase 1 dose-escalation study (NCT02122146) was initiated to evaluate the safety and tolerability of PF-06664178 in patients with advanced solid tumors. However, patients treated with PF-06664178 showed no objective responses, and the drug exhibited toxicity at high doses with only modest antitumor activity 32. Seven patients experienced at least one dose-limiting toxicity (DLT), such as neutropenia or rash. Owing to the potential lack of a therapeutic window, further development of PF-06664178 was discontinued. The excess toxicities of PF-06664178 are thought to be attributed to its highly potent payload and stable linker.

**SHR-A1921.** SHR-A1921, developed by Hengrui, is a novel Trop2-directed ADC with an optimized DAR of 4. It comprises a proprietary IgG1 mAb conjugated to DNA topoisomerase I inhibitor via a tetrapeptide-based cleavable linker 33. The payload, named SHR9265, is a novel exatecan derivative characterized by great liposolubility and cellular permeability. Compared to SG and SKB264, SHR-A1921 demonstrated stronger binding affinity, improved plasma stability, a more effective bystander effect, superior *in vivo* efficacy, and a longer half-life. Preliminary data from a FIH phase 1 study (NCT05154604) of SHR-A1921 showed an ORR of 33.3% and a disease control rate (DCR) of 80% in patients with pretreated advanced cancer 34. In all dose cohorts, the most frequent treatment-related adverse events (TRAEs; occurring in ≥30% of participants) included nausea (71.1%), stomatitis (65.8%), anemia (42.1%), and vomiting, decreased appetite, decreased weight, and rash, each affecting 36.8% of patients. Additional ongoing clinical trials (NCT05765032, NCT06394492, and NCT05594875) aim to assess the safety and antitumor activity of SHR-A1921, either alone or in combination with other anticancer agents, in patients with advanced solid tumors.

**ESG-401 (STI-3258).** ESG-401 consists of a humanized Trop2-directed IgG1 mAb, conjugated to SN-38 via a highly stable and cleavable linker, featuring a DAR of 8. Upon binding to Trop2-expressing tumor cells, ESG-401 could release its payload to inhibit cell proliferation after rapid endocytosis. Preclinical models showed minimal free toxins and high enrichment in tumor tissues. Furthermore, ESG-401 exerted potent *in vivo* inhibitory effects in various Trop2-expressing tumor models at a low effective dosage and with prolonged tumor growth suppression. Regarding the safety profile, no off-target or off-tumor toxicities were observed in non-human primates that repetitively received ESG-401. The preliminary results of a FIH phase 1 trial (NCT04892342) with ESG-401 demonstrated good safety and tolerability with promising signals of efficacy in heavily pretreated patients with locally advanced or metastatic solid tumors 35. The most frequently reported TRAEs included leukopenia (80%), neutropenia (69%), anemia (66%), fatigue (54%), nausea (51%), and vomiting (46%). No instances of Grade 3 thrombocytopenia, diarrhea, skin rash, or oral mucositis were observed. No cases of ILD were detected. Further clinical evaluations are necessary to determine its therapeutic potential.

**LCB84.** LCB84, developed by LegoChem Biosciences, is a next-generation ADC designed for the treatment of solid tumors that express Trop2. It consists of monomethyl auristatin E (MMAE) as the payload and a humanized IgG1 mAb (Hu2G10) that can selectively target ADAM10-activated Trop2 on transformed tumor cells 36. LegoChem Biosciences engineered LCB84 with a plasma-stable cleavable linker to allow for efficient and traceless payload release in a cancer-specific manner 37. Compared with SG and Dato-DXd, LCB84 exhibited significantly enhanced *in vivo* antitumor activity in multiple CDX models, such as those for TNBC, NSCLC, and pancreatic ductal adenocarcinoma (PDAC). Additionally, toxicity testing indicated that LCB84 was well-tolerated with a therapeutic index (TI) of approximately 30 for single dosing and 40 for repeat dosing in cynomolgus monkeys. Encouraged by positive preclinical results, a FIH phase 1/2 clinical trial (NCT05941507) has commenced to evaluate the efficacy of LCB84 as both a single agent or in combination with programmed cell death 1 (PD-1) inhibitor for the treatment of advanced solid tumors.

While ADCs hold immense therapeutic promise in Trop2-positive tumors, actualizing this potential demands overcoming several critical challenges, including intratumor and intratumor heterogeneity, the risks of TRAEs, and drug resistance. The physicochemical and biophysical properties of an ADC are profoundly influenced by its components, including the naked antibody, chemical linker, cytotoxic payload, and conjugation chemistry 38. To develop next-generation Trop2-targeted ADCs with an ideal efficacy-safety balance, comprehensive optimization of each component is necessary. Notably, Trop2 is expressed on both tumor cells and certain nonmalignant tissues, leading to unavoidable on-target off-tumor toxicities in many clinical trials, resulting in some being discontinued. Therefore, improving tumor-specific binding activity is a crucial objective for developing novel Trop2-targeted ADCs. Moreover, the identification of biomarkers and thorough patient stratification might enhance the clinical outcomes of Trop2-directed ADC therapies. Beyond the traditional ADC format featuring small-molecule drug payloads, alternative conjugation strategies like immunoconjugates and nanoparticles are emerging in preclinical development 39, 40. Liu *et al.* engineered a Trop2-targeted tetrakis-ranpirnase conjugate, composed of hRS7 mAb fused with four copies of ranpirnase 41. This novel IgG-Rap immunoRNase provided a significant survival benefit in TNBC mouse models.

### Trop2-directed monoclonal antibodies

Although Trop2-targeted ADCs have shown clinical benefits, their broader utility is still limited by issues such as off-target payload-mediated toxicities 32, 42-44. Consequently, there is a need to develop safer and more effective targeted agents that leverage the biological functions of Trop2 itself. Recent advances in structural biology have uncovered the unique oligomerization properties of Trop2, which are critical in oncogenic signaling pathways and associated with poor prognosis 45-47. Furthermore, the large extracellular domain (ECD) makes it an ideal therapeutic target for Trop2-targeted drugs that are designed to block its oncogenic activity 48. As a result, the development of functional Trop2-directed mAb has become a focal point of recent research. In the following discussion, we will explore recently identified anti-Trop2 mAbs that exhibit potent antitumor activity and possess unique MOAs. Additional mAbs in preclinical development are listed in Table 2.

**Figure 2:**
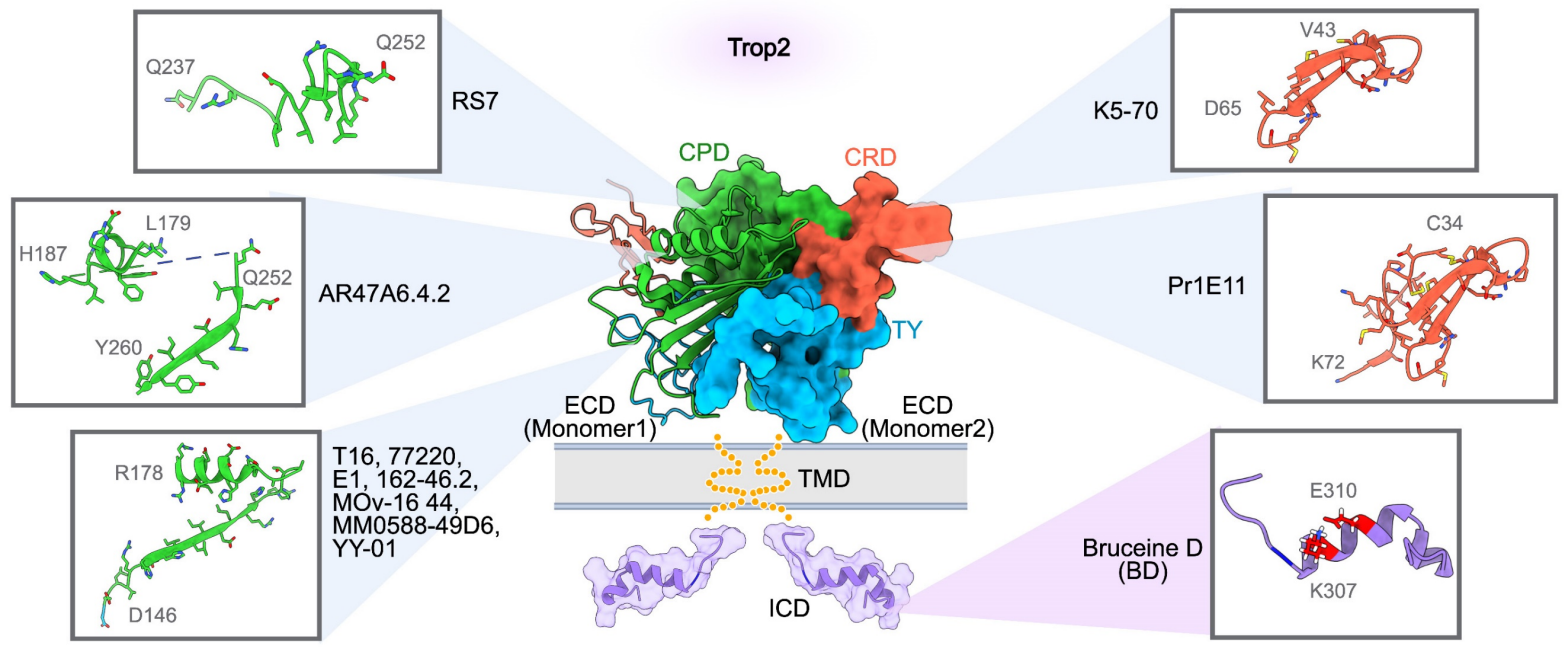
** Binding sites of Trop2-targeted antibodies and small molecular inhibitors.** A composite model of full-length Trop2 dimer, projected based on the structures of individual functional modules. PDB identifier 7E5N for ECD, and PDB identifier 2MVK for ICD. RS7 recognizes Q237-Q252 of CPD. AR47A6.4.2 recognizes L179-H187 and Q252-Y260 of CPD. T16, 77220, E1, 162-46.2, MOv-16, MM0588-49D6, and YY-01 recognize D146-R178 of CPD. K5-70 recognizes V43-D65 of CRD. Pr1E11 recognizes C34-K72 of CRD. The only small molecule inhibitor, Bruceine D (BD), binds to the key sites K307 and E310 of Trop2-ICD.

RS7-3G11 (RS7), the antibody component of SG, is an extensively studied anti-Trop2 mAb from early research. Stein *et al.* obtained RS7 using the hybridoma technique 49, 50. On its own, RS7 demonstrates strong internalization activity but lacks therapeutic efficacy 11, 51. In 2021, Sun and colleagues constructed domain-substituted Trop2 mutants to map the epitopes of RS7 46. Binding analysis showed that RS7 recognizes a linear polypeptide, Q237-Q252, within the cysteine-poor domain (CPD) of Trop2-ECD (Figure 2). This stretched loop is far from the interfaces of the Trop2-ECD dimer, suggesting that RS7 is unlikely to disrupt the oligomeric assembly of Trop2. This could explain why RS7, in its unconjugated form, does not exert a suppressive effect on Trop2-positive tumors. Similarly, a group of mAbs, such as T16, 77220, E1 52, 162-46.2, MOv-16 53, MM0588-49D6, and YY-01 54 exhibit limited therapeutic effects.

This limitation is partly because their binding epitopes (D146-R178) are distant from the interaction region essential for Trop2 dimerization. These results suggest that the CPD region of Trop2 may comprise multiple immunodominant epitopes, enabling easy screening of numerous antibodies that, however, lack therapeutic functions. Additional research has indicated that these immunodominant epitopes appear to be equally accessible in both tumors and normal cells, raising concerns that Trop2-directed mAbs targeting these epitopes may cause unmanageable off-tumor toxicity 6, 53, 55. Consequently, the approach of targeting tumor-specific epitopes on Trop2 seems more promising.

Alberti's team identified the cleavage of Trop2 by ADAM metallopeptidase domain 10 (ADAM10) as a critical switch activating tumor growth and metastasis 52. The proteolysis of Trop-2 at R87-T88, mediated by ADAM10, could induce a profound structural rearrangement, potentially exposing binding sites that are typically inaccessible 56. To discover mAbs sensitive to these tumor-specific sites, Alberti *et al.* employed a deletion mutagenesis strategy to remove the immunodominant regions of Trop2 and then selected mAbs by multiple methods 36. The 2G10 mAb, which selectively recognizes cleaved Trop2 in transformed cells, was obtained and exhibited inhibitory activity on tumor cell proliferation *in vitro*. Humanized 2G10 (Hu2G10) binds cancer-specific, cleaved/activated Trop2 with a high affinity (K_D_ < 10^-12^ M), and effectively suppresses the growth of Trop2-positive tumors *in vivo*, including breast, colon, and prostate cancers 36, 57. The ADC version of Hu2G10, known as LCB84, is being evaluated in an FIH phase 1/2 clinical trial (NCT05941507) 37. Given that the combination of two mAbs, binding non-overlapping epitopes, could offer complementary MOAs and lead to synergistic therapeutic efficacy, Alberti's team explored the combined *in vivo* activity of 2G10 and 2EF-an anti-Trop2 mAb recognizing N-terminal epitopes of Trop2-ECD with proven antitumor activity across multiple tumor models 58. Analysis of Trop2-expressing CDX models demonstrated that 2EF can serve as a potent synergistic partner, significantly boosting the *in vivo* tumor-killing effect of 2G10. Furthermore, Hu2G10 and humanized 2EF (Hu2EF) were well-tolerated from 5 to 10 mg/kg, with no evidence of systemic toxicity observed in cynomolgus monkeys 59. No significant neurological, respiratory, digestive, or urinary symptoms, including vomiting, diarrhea, and anorexia, nor any biochemical or hematological toxicities were observed during the 28-day period. These findings support the therapeutic potential of a tumor-selective strategy targeting the activated Trop2 in cancer cells.

ARIUS Research utilized the FunctionFIRST platform to generate a mAb, AR47A6.4.2, which targets two liner epitopes (L179-H187 and Q252-Y260) within Trop2-CPD (Figure 2). AR47A6.4.2 can inhibit Trop2 signaling and mediate the downregulation of mitogen-activated protein kinase (MAPK) pathway in response to serum stimulation 60. It also induces complement-dependent cytotoxicity (CDC) in human pancreatic cells. The dual MOAs empower AR47A6.4.2 to inhibit tumor growth in human models of breast (90%, p<0.00001), colon (60%, p<0.001), and prostate (60.9%, p<0.001) cancer. In addition, an enhanced therapeutic efficacy was observed when AR47A6.4.2 was combined with Gemcitabine, a chemotherapy drug for pancreatic cancer, in the pancreatic tumor model 61. Notably, the Q252-Y260 epitope is located at the interfaces of both the *cis-*dimer and the *trans-*dimer of Trop2, implying that AR47A6.4.2 may inhibit tumor growth by preventing Trop2 dimerization 46, 48.

Crystallographic studies of Trop2-ECD have revealed that the N-terminal region, especially the cysteine-rich domain (CRD), is essential for the tetrameric assembly 45, 46. Therefore, targeting the epitopes within the CRD could influence the dynamic behavior of Trop2, potentially disrupting its activation. Pr1E11, an early reported CRD-targeted mAb, showed low internalization activity but great cell surface retention in Trop2-expressing tumor cells 54, 62. While Pr1E11 did not show an antiproliferative effect *in vitro*, it inhibited tumor growth more effectively than AR47A6.4.2 *in vivo*, aided in part by its potent antibody-dependent cellular cytotoxicity (ADCC). Further research is needed to ascertain if the CRD-binding of Pr1E11 affects the oligomeric state of Trop2 on the tumor cell surface, a crucial factor in Trop2-mediated tumor progression. K5-70, developed by Chiome Bioscience (patent CN107236043B), is another CRD-directed mAb. Chemically linked peptides on scaffolds technology (CLIPS) elucidated that the key epitope of K5-70 is located on V43-D65 polypeptide within Trop2-CRD (Figure 2). This binding region overlaps with the tetramerization interface of Trop2, hinting that K5-70' potential to disrupt the Trop2 clustering on the tumor cell surface and subsequently interfere with its oncogenic signaling pathways. Potent inhibitory effects of K5-70 were observed *in vivo* across different tumor models such as SW480, DU-145, and PK-59. Additionally, K5-70 effectively suppressed a recurrent tumor model previously treated with irinotecan hydrochloride. These findings confirm the therapeutic potential of CRD-targeted mAb in Trop2-positive tumors.

Besides the aforementioned antibodies with well-defined epitopes, many antibodies with unknown binding sites also exhibit promising antitumor properties. Zhou and colleagues recently reported a novel Trop2-directed mAb, IMB1636, notable for its high affinity and specificity for Trop2-expressing tumors 63. IMB1636 demonstrated potent internalization activity and sustained tumor-targeting capability* in vivo*, with an impressive retention of 264 hours. The humanized form of IMB1636 (hIMB1636) recognizes non-competitive binding sites compared to hRS7 and retains robust antitumor activity in MDA-MB-468 xenograft tumors. The promising *in vivo* antitumor effects are attributable to at least three MOAs: ADCC, the inhibition of cancer cell proliferation and migration, and the induction of cell cycle arrest and apoptosis. Sun *et al.* further constructed an ADC version of hIMB1636 (hIMB1636-MMAE), using a Valine-Citrulline linker to conjugate mAb with three to four molecules of MMAE 64. Not only does hIMB1636 retain the MOAs of its parent antibody, but it also achieves enhanced tumor inhibition via bystander effect in pancreatic cancer. TANAKA *et al.* generated an anti-Trop2 mAb TrMab‑6 via the Cell‑Based Immunization and Screening (CBIS) method 65. TrMab‑6 strongly triggers ADCC and CDC, leading to pronounced tumor reduction in breast cancer mouse xenograft models. Beyond the conventional IgG antibodies, Trop2-targeted Fab and nanobodies (Nb) may also serve as therapeutic agents in Trop2-expressing tumors. A fully human Fab against Trop2, isolated from a phage library by Lin and colleagues, inhibits breast cancer growth *in vitro* and *in vivo* by inducing the downregulation of Bcl-2 and upregulation of Bax in Trop2-expressing tumor cells 66. This Fab was subsequently used to construct an anti-Trop2 IgG mAb, which inhibits tumor cell growth, migration, and invasion *in vitro*, and suppresses tumor proliferation in ovarian cancer xenografts 67. Hu *et al.* recently described a Trop2-targeted nanobody, N60, that is capable of inhibiting tumor cell proliferation and migration 68.

Overall, antibodies targeting Trop2, particularly those that recognize tumor-specific Trop2, have demonstrated promising therapeutic potential and safety profiles. However, a key challenge hinders their clinical translation: the fundamental relationship between antibodies' binding epitopes and MOAs that contribute to its antitumor effects. It should also be noted that most reported anti-Trop2 mAbs primarily target the CRD and CPD domains, with fewer directed against the thyroglobulin type-1 (TY) domain (Figure 2). Structurally, the TY region is extensively involved in the oligomeric assembly of Trop2 and includes the conserved sites crucial for tumor-specific proteolytic cleavage. Thus, it is reasonable to speculate that targeting TY may interfere with Trop2-mediated intracellular and intercellular communication, potentially hindering the progression of Trop2-positive tumors. Consequently, the discovery of novel mAbs targeting the TY region could offer an alternative therapeutic approach for managing Trop2-positive tumors. Given the widespread expression of Trop2 in normal epithelial tissues, employing non-human primate (NHP) models to assess the safety profile is essential for the preclinical development of the next generation of Trop2-targeted antibodies.

### Multispecific agents

Drug resistance and tumor heterogeneity are well-known factors that can limit the clinical outcomes of targeted therapies directed toward a single antigen 8. To address this challenge, the development of multispecific agents has gained attention as a technique allowing concurrent binding to two or multiple distinct targets, either molecules or cells. A variety of emerging multispecific agents associated with Trop2, such as bispecific antibodies, trispecific antibodies, and bispecific ADCs, have been evaluated in preclinical settings (Table 3). Below, we discuss their MOAs, antitumor efficacy, and limitations.

#### Bispecific antibodies

**Figure 3:**
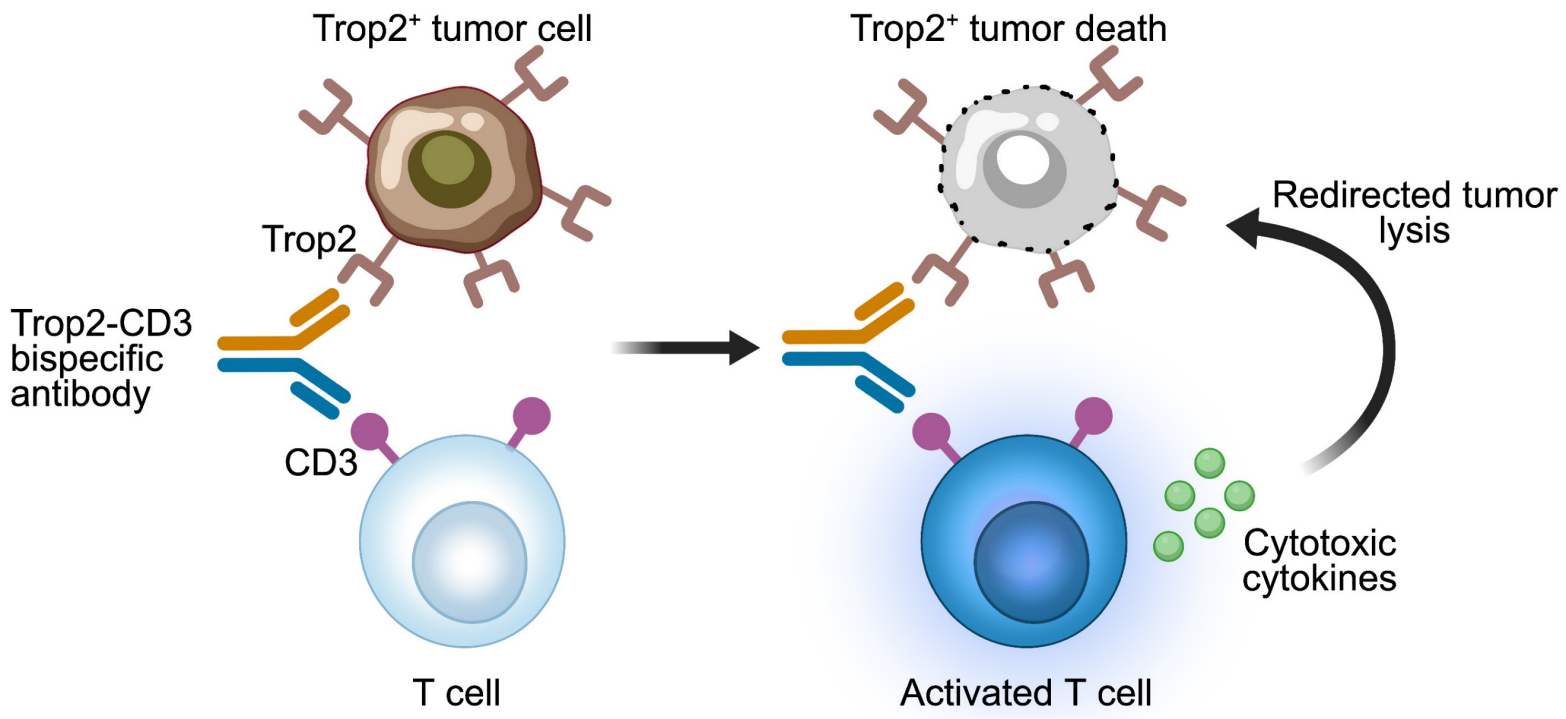
** Mechanisms of action of Trop2-CD3 bispecific T cell engagers.** The Trop2-CD3 bispecific antibody binds to CD3 on T cells and Trop2 antigen on tumor cells. The activated T cells release cytotoxic cytokines, resulting in the lysis of Trop2-positive tumor cells. The image was created with *MedPeer.cn*.

Various formats of bispecific antibodies have been engineered to enhance the antitumor activity and tumor specificity in Trop2-positive solid tumors. The early reported anti-Trop2 bispecific antibodies are bispecific T cell engagers (BiTEs) that use CD3 as a target to redirect T cells towards Trop2-expressing tumor cells (Figure 3). An early BiTEs candidate, (E1)-3s, comprised an anti-CD3 single-chain variable fragment (scFv) fused to a stabilized dimer of a Trop2-targeted Fab (derived from hRS7) via Dock-and-Lock (DNL) technique 69. Surface plasmon resonance analysis showed that (E1)-3s maintained high Trop2-binding affinity (K_D_ = 1.03 ± 0.19 nM). Bidirectional trogocytosis and functional immunologic synapses induced by (E1)-3s were observed between T cells and target cells. (E1)-3s effectively mediated T-cell activity, leading to the potent lysis of target tumor cells by T-cell. Promising T-cell-mediated killing of Trop2-expressing pancreatic and gastric cancers triggered by (E1)-3s were observed *in vitro* and *in vivo*. Moreover, this T-cell redirection against solid cancers was significantly enhanced by interferon-α (INFα). Furthermore, combining (E1)-3s with an antagonistic anti-PD-1 antibody boosted the antitumor potency of T-cells, leading to enhanced cell death in 3D spheroid cultures and prolonged the survival in mice with MDA-MB-231 tumors 70. These preclinical findings highlight the potential benefits of combining T-cell-redirecting bispecific antibodies with agents that counteract T-cell inhibition in the tumor microenvironment (TME), potentially improving the effectiveness of immunotherapy in patients with solid tumors.

Liu and colleagues engineered an alternative Trop2-targeted BiTE, named F7AK3, designed to bind both CD3 and Trop2, with a predominant affinity for Trop2 71. This design aims to prevent non-specific peripheral activation of T-cells and to localize T-cells within the tumors. The tetravalent F7AK3 is composed of an anti-CD3 scFv fused to the Fc region of a human anti-Trop2 IgG4. The *in vitro* analysis showed that F7AK3 triggered T cell-mediated cytotoxicity against several Trop2-expressing tumor cell types, with the level of activation depending on Trop2 antigen expression. In TNBC xenograft models, F7AK3 was shown to inhibit tumor growth via multiple MOAs. First, F7AK3 treatment resulted in a significant expansion of human immune cells within the spleens and tumors. Second, the treatment enhanced the expression of CD69 and PD1 in both CD8^+^ and CD4^+^ T cells.

Third, F7AK3 induced significant upregulation of IL-2 and Granzyme B in CD4^+^ T cells. Considering the increased risk of cytokine release syndrome due to excessive T cell activation, Wang's team constructed a Trop2-CD3 BiTE with a reduced affinity for CD3, called Trop2/CD3b 72. Trop2/CD3b consists of an anti-CD3 scFv (from SP34) inserted into the heavy chain of an anti-Trop2 mAb (from Pr1E11). Crucially, Trop2/CD3b elicited a notably lower level of Th1 cytokines production but still effectively maintained tumor cell cytotoxicity *in vitro* and tumor growth suppression *in vivo*. To reduce the risk of on-target off-tumor effects, Arbele developed a novel Trop2-CD3 BiTE with weaker binding affinity to cells expressing Trop2 73. Comparable to F7AK3 and (E1)-3s, this Trop2-CD3 BiTE was created by fusing a humanized anti-Trop2 mAb with an anti-CD3 scFv. Unlike F7AK3 and (E1)-3s, which both bind tightly to Trop2 antigen, the modified Trop2-CD3 BiTE has a lower affinity with a K_D_ of 214 nM. This is achieved by altering a complementarity-determining region (CDR) on the heavy chain of the anti-Trop2 mAb component. This bispecific antibody candidate merits further exploration, with additional toxicity assessments in non-human primates. These results suggest that optimizing a Trop2-CD3 bispecific antibody requires careful calibration of safety and efficacy, aiming to achieve tumor-specific cytotoxicity without eliciting excessive T-cell activation. Besides the Trop2-CD3 bispecific antibodies, additional bispecific formats like Trop2-HER2, Trop2-PD-L1, and Trop2-CD28 are currently under preclinical investigation (Table 3).

#### Trispecific antibodies

To circumvent the potential for on-target off-tumor toxicities leading to treatment failure, HARPOON Therapeutics has developed a Trop2-targeted ProTriTAC^™^ (Protease-activated Tri-specific T cell Activating Construct), a prodrug designed for selective activation within tumor tissues. This trispecific T cell engager comprises three antibody-derived binding domains on a single polypeptide chain: one to extend half-life through albumin binding, a second for T cell engagement via CD3 binding, and a third targeting Trop2-positive tumor cells 74. The anti-albumin domain, equipped with a masking moiety and a protease-cleavable linker, renders the prodrug inactive by preventing the nearby anti-CD3 domain from binding to T cells. Once tumor-associated proteases cleave the linker, the anti-albumin domain and the masking moiety are detached, uncovering a highly active drug that redirects T cells to lyse tumor cells. *In vitro* experiments revealed that the protease-activated T cell engager exhibits over a 1000-fold increase in binding affinity to primary human T cells and approximately a 100-fold enhancement in T cell cytotoxicity compared to the masked prodrug. In human T cell-reconstituted immunodeficient mice, this anti-Trop2 ProTriTAC displayed potent antitumor effects across various xenograft models, such as HPAFII (pancreatic cancer), CAL27 (head and neck cancer), and HCC70 (breast cancer), with complete tumor regression at doses as low as 30 µg/kg. ProTriTAC showed favorable pharmacokinetics and was well-tolerated up to 180 µg/kg in cynomolgus monkeys. Boehringer Ingelheim has recently patented a novel trispecific T-cell engager that targets Trop2, CD3, and cadherin-17 (CDH-17). The trispecific Trop2/CD3/CDH-17 binding molecule effectively mediated the lysis of Trop2^+^ CDH-17^+^ cells *in vitro* and induced significant tumor regression *in vivo* in human xenograft mouse models (HPAF-II) reconstituted with human T cells. Additional clinical investigation is required to assess the efficacy and safety of these FIC conditionally active, Trop2-directed trispecific antibodies.

#### Bispecific ADCs

Bispecific ADCs are emerging targeted agent that simultaneously targets two different antigens. Compared to traditional ADC drugs, bispecific ADCs may present several benefits. First, they can identify and eliminate a wider range of tumor cells, including those in heterogeneous tumor tissues. Second, by selecting optimal targets, bispecific ADCs could enhance tumor-specific binding, given the restricted expression of the target antigens on nonmalignant cells, potentially boosting payload delivery while minimizing toxicities in healthy tissues 8. Moreover, the simultaneous targeting of multiple antigens or cells could exert a synergistic effect, which may not be attenable by traditional ADCs recognizing a single antigen. Considering these potential advantages, researchers have developed a variety of anti-Trop2 bispecific ADCs, predominantly choosing tumor-associated kinases as their secondary targets.

EGFR is overexpressed in numerous solid tumors and has become a well-established therapeutic target. However, drug resistance and low cytotoxicity remain challenges. Trop2 and EGFR are co-expressed in various solid tumors, such as those of the head and neck, esophagus, lungs, and pancreas, suggesting the potential for a combined therapeutic approach to benefit a diverse set of tumors. Li and colleagues engineered a FIC Trop2-EGFR bispecific ADC, named DM001, using a protease-cleavable linker to conjugate antibody with MMAE 75. In tumor cells expressing both Trop2 and EGFR, DM001 exhibited comparable internalization and tumor-killing efficacy to that of its parent anti-Trop2 and anti-EGFR mAbs. Furthermore, DM001 demonstrated enhanced selectivity and potency in binding and killing cells with co-expression of Trop2 and EGFR, in comparison to those expressing single targets. The potent *in vitro* antitumor effect of DM001 is attributable to at least two MOAs: the induction of cell cycle arrest and the elevation of apoptosis rates in an antigen-dependent manner. Notably, DM001 outperformed SG and its parental ADCs in a range of CDX and PDX models, including those for lung and pancreatic tumors. The prior efficacy of DM001 in PDX models indicated that it may be more effective in targeting heterogeneous tumors, which better mimics the TME of patients. Beijing Biocytogen has also developed a Trop2-EGFR bispecific ADC, called BCG011, which is currently undergoing preclinical evaluation.

HER2, another well-established therapeutic target, is often found co-expressed with Trop2 across various solid tumor types, including NSCLC, breast cancer (BC), gastric cancer (GC), and bladder cancer. Shang *et al.* recently reported a FIC Trop2-HER2 bispecific ADC (YH012), which consists of a bispecific antibody conjugated with MMAE via a protease-cleavable linker 76. Compared to its parental anti-Trop2 or anti-HER2 mAbs, YH012 exhibited enhanced affinity, internalization activity, and tumor selectivity *in vitro*. The *in vivo* studies demonstrated that YH012 effectively suppressed tumor growth in a range of CDX and PDX models, including NSCLC, BC, GC, and pancreatic cancer. Furthermore, YH012 outperformed benchmark antibodies in reducing tumors in both HER2-high and HER2-low-expressing xenograft models, suggesting a robust and expansive therapeutic impact. As a member of the EGFR family, human epidermal growth factor receptor 3 (HER3) plays a key role in regulating the proliferation of epithelial cells 77. HER3 is a potent activator of the phosphatidylinositide-3 kinase (PI3K)/Akt survival pathway, which is vital for tumor expansion. Alphamab Oncology has recently developed a Trop2-HER3 bispecific ADC, named JSKN016, that was engineered using a Glycan-specific conjugation platform. JSKN016 can induce apoptosis in tumor cells that express either Trop2 or HER3. Additionally, it can penetrate the cell membrane of antigen-negative tumor cells, exerting a bystander effect that helps to inhibit tumor growth through topoisomerase I inhibitor mechanism. The clinical investigation of JSKN016 for treating advanced malignant solid tumors has been approved by China's Center for Drug Evaluation (CDE).

Protein tyrosine kinase-7 (PTK7) exhibits overexpression in breast cancer, with notably higher levels found in TNBC than in non-TNBC. Overexpressed PTK7 is associated with the progression of TNBC and a worse prognosis 78. Yao and colleagues generated a novel Trop2-PTK7 bispecific ADC (BCG033) composed of a fully human bispecific antibody conjugated with MMAE 79. Preliminary data indicated that BCG033 had favorable tumor selectivity and exhibited potent antitumor efficacy in both TNBC and non-TNBC CDX models, which co-expressed Trop2 and PTK7. Beyond human IgG1-based scaffolds, the nanobody format has been utilized in constructing bispecific ADCs. HuNb_TROP2-HSA_-MMAE is a nanobody-drug conjugate that simultaneously targets Trop2 and human serum albumin (HSA) 80. The humanized anti-Trop2 nanobody was fused to an anti-HSA nanobody and conjugated to MMAE by a cleavable linker with a DAR of 1. HuNb_TROP2-HSA_-MMAE retained high affinity and binding specificity for Trop2 and HSA. The potent internalization activity of HuNb_TROP2-HSA_-MMAE facilitated its involvement in lysosomal trafficking, leading to the release of cytotoxic payload. Its tumor-killing efficacy in Trop2-expressing pancreatic cancer is attributed to caspase-3/9-induced cell apoptosis. The *in vivo* studies using pancreatic cancer CDX models revealed that doses of HuNb_TROP2-HSA_-MMAE at 0.2 mg/kg and 1 mg/kg achieved notable antitumor effects, with complete tumor eradication achieved at a dose of 5 mg/kg.

### CAR-T and CAR-NK

Chimeric antigen receptors (CARs) are engineered constructs that combine the targeting precision of antibodies with the effector function signaling of T cells, forming a pivotal subset of cellular immunotherapies. While CAR-T therapies have received FDA approval for treating hematological malignancies, challenges with the directed migration and infiltration of T cells into the TME impede their effectiveness against solid tumors. Nonetheless, some Trop2-specific CARs have demonstrated promising efficacy in preclinical or early clinical stages (Table 4). Cancer Hospital Chinese Academy of Medical Sciences has developed a Trop2 CAR-T that has been evaluated with advanced solid tumors in a phase 1/2 clinical trial (NCT06082557). For cellular preparation, peripheral blood mononuclear cells (PBMCs) are utilized. Using the blood cell apheresis technique, T cells expressing PD-1 are separated from the peripheral blood and transduced with a lentivirus carrying both an "enhanced receptor" and a Trop2-CAR. These resultant T60c cells are then administered through a single intravenous infusion. In 2019, Zhao and colleagues engineered a bispecific Trop2/PD-L1 CAR-T therapy with a potent cytotoxic effect against GC 81. These bispecific CAR-T cells induced higher cytokine levels *in vitro* and showed more potent inhibitory activity *in vivo* compared to monospecific CAR-T cells. However, as both Trop2 and PD-L1 are expressed in normal tissues, further research is necessary to determine whether the bispecific strategy might lead to off-tumor toxicities.

In addition to T cells, alternative immune cell types, specifically natural killer (NK) cells, have been genetically engineered to express chimeric antigen receptors (CAR-NK), redirecting their cytotoxic abilities toward solid tumors. In 2023, the M.D. Anderson Cancer Center initiated two clinical studies (NCT05922930 and NCT06066424) to evaluate Trop2 CAR-engineered IL15-transduced cord blood-derived NK cells in patients with platinum-resistant ovarian cancer, mesonephric-like adenocarcinoma, and pancreatic cancer. Poorebrahim and colleagues recently described an NK cell line designed for constitutively expressing the human CD3 and CD8 genes. Furthermore, the NK cell was engineered with co-expression of a human papillomavirus type 16 oncoprotein E7-reactive T cell receptor (E7-TCR) and a costimulatory Trop2-CAR 82. The resultant E7-TCR/Trop2-CAR NK cells demonstrated increased antigen-specific activation and exhibited enhanced cytotoxicity against tumor cells than NK cells equipped with only the E7-TCR. The inclusion of a costimulatory Trop2-CAR indeed synergizes with E7-TCR in NK cells, enhancing their signaling capabilities and targeted cytotoxic response against antigen-presenting tumor cells. Beyond varying immune cell types, researchers are refining Trop2-CAR therapies by optimizing expression methodologies. Myeloid Therapeutic has designed a novel Trop2-CAR, named MT-302, engineered to be expressed within the myeloid compartment. Treatment with MT-302 has shown effectiveness as a monotherapy in a Trop2-positive TNBC model, highlighting the efficacy of the engineered myeloid cells without the need for T-cell involvement. A FIH phase 1 clinical study has been initiated to assess the safety, tolerability, and recommended phase 2 dosing (RP2D) of MT-302 in patients facing advanced or metastatic epithelial tumors.

### Cancer vaccines targeting Trop2

Cancer vaccines are formulated to activate a patient's immune system, empowering it to detect and eliminate tumor cells through the induction of B cells and elicitation of CD8^+^/CD4^+^ T cell responses against tumor-specific antigens 83. Several Trop2-specific cancer vaccines are currently under development at the preclinical stage using different platforms.

In 2011, Cubas and colleagues used virus-like particles (VLPs) platform to generate murine Trop2 (mTrop2) VLP by incorporating full-length mTrop2 proteins into the membrane envelope of simian immunodeficiency virus (SIV) VLPs 84. Vaccination with chimeric mTrop2 VLPs can break self-tolerance and initiate robust cellular and humoral immune responses, markedly enhancing the presence of CD4^+^ and CD8^+^ T cells, along with NK and natural killer T (NKT) cells, within tumor tissues. The tumor-infiltrating lymphocytes (TILs) elicited by this vaccine not only maintained an activated effector phenotype but also modulated numerous suppressive elements within the TME, including regulatory T (Treg) cells, myeloid-derived suppressor cells (MDSCs), and cytokines like Interleukin 10 (IL-10) and transforming growth factor-β (TGF-β). These MOAs translated into a noteworthy decrease in tumor growth and an increased survival rate for mice bearing Trop2-expressing pancreatic cancer. Combining mTrop2 VLP vaccination with gemcitabine therapy, a prevalent chemotherapeutic agent for pancreatic cancer, resulted in a remarkable improvement in the survival rates of mice, surpassing the outcomes achieved with either treatment alone. These findings suggest a potential immunotherapeutic strategy for Trop2-positive tumors using VLPs as a vector to provoke a full immune response against Trop2 antigen, leading to the activation of a diverse range of immune effector cells and the modulation of crucial immunosuppressive factors within the TME. In 2018, Xi *et al.* constructed a Trop2-CD40 ligand VLPs (Trop2-CD40L VLPs) employing a human immunodeficiency virus (HIV) gag‑based strategy 85. The *in vivo* analysis revealed that the incorporation of CD40 ligand significantly boosted the immunogenicity of Trop2-CD40L VLPs against lung cancer, resulting in more effective tumor control and an increased survival rate in Lewis (Trop2^+^) tumor-bearing mice. Potent humoral and cellular immune responses triggered by Trop2-CD40L VLPs demonstrated the adjuvant potential of CD40L in VLP‑based cancer vaccines.

**Figure 4:**
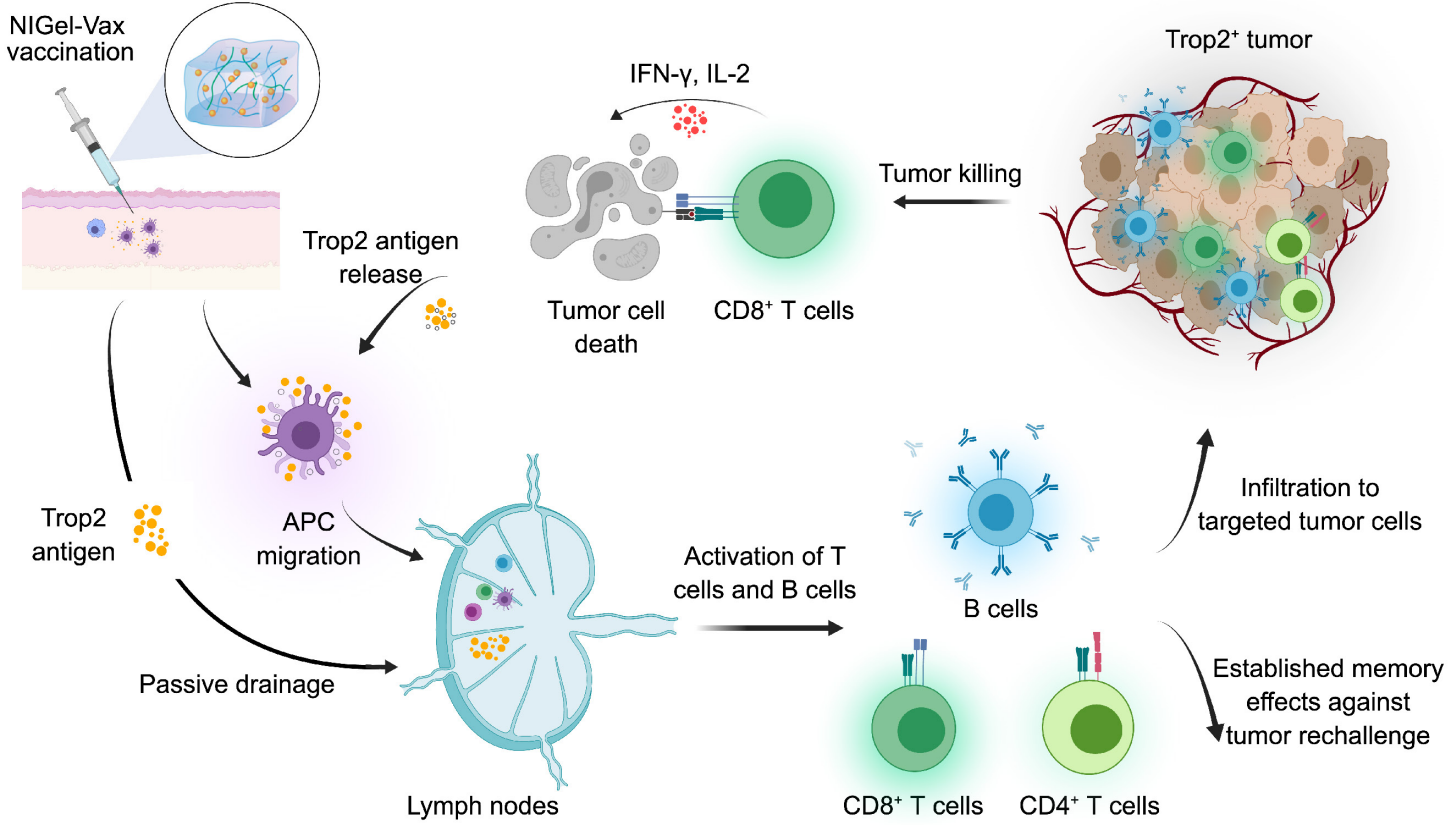
** Mechanisms of action of a Trop2-targeted Nano-in-Gel Vaccine (NIGel-Vax) for treating Trop2-expressing tumors.** The Trop2 antigen is mixed with PEI-4BImi-Man adjuvant to form nanoparticles, then incorporated into an injectable hydrogel to construct the NIGel-Vax. After subcutaneous vaccination, the Trop2-containing nanoparticles are sustainably released and are transported to lymph nodes by dendritic cells (DCs) or through passive drainage. Subsequently, activated B cells produce antigen-specific antibodies, and activated T cells migrate to the TME, leading to direct tumor killing or tumor cell apoptosis. Furthermore, NIGel-Vax induces the generation of central memory and effector memory T cells, primed for potential tumor rechallenge. Immunogenic dead tumor cells can release Trop2 antigens and danger signaling molecules, amplifying the intensity and breadth of the subsequent immune response. The image was created with *MedPeer.cn*.

To achieve the precise spatial and temporal control of vaccine kinetics, Liu and colleagues recently designed a novel one-shot injectable nano-in-gel vaccine (NIGel-Vax) for postoperative breast cancer therapy 86. The Trop2 antigens were combined with a polyethylenimine-benzoimidazole-4-carboxylic acid-2-isothiocyanatoethyl-α-Dmannopyranoside (PEI-4BImi-Man) adjuvant, known for its inherent immunostimulatory properties and the capacity to target dendritic cells (DCs) via mannose receptors. Subsequently, the resulting nanoparticles were encapsulated in a dynamic covalent hydrogel with 4-arm-PEG-ONH_2_ and oxidized dextran (ODEX) via oxime-linkages. Using a 4T1 postoperative TNBC recurrence model, NIGel-Vax accomplished a 96% tumor suppression rate and a 50% cure rate due to enhanced immune activation. Furthermore, the injectable nano-in-gel vaccine system could establish a long-lasting immune memory effect, providing sufficient protection against tumor rechallenges (Figure 4). Overall, while Trop2-specific cancer vaccines can elicit immunogenicity and improve long-term survival in mouse models, their efficacy and safety profile must be validated through clinical trials.

### Small molecular inhibitor

Antibodies are typically characterized by their high specificity and affinity for cell surface targets, which often restricts their scope of action. Small molecule agents are capable of inhibiting tumor growth by targeting an extensive array of extracellular and intracellular proteins, positioning them as a viable alternative for targeted cancer therapy. Bruceine D (BD) is currently the sole small molecule inhibitor known to exhibit antitumor activity in Trop2-positive tumor cells. Tang and colleagues discovered BD's inhibitory effects using techniques such as cell membrane chromatography (CMC) and cellular thermal shift assay (CETSA) 87. Molecular docking and site-directed mutagenesis studies revealed that BD recognized the intracellular domain (ICD) of Trop2, identifying K307 and E310 as key binding sites (Figure 2). The *in vitro* and *in vivo* studies focusing on Trop2-dependent inhibitory activity further confirmed the target specificity of BD. Mechanistically, BD suppressed Trop2-induced cancer metastasis by interfering with the Trop2/β-catenin positive feedback loop, wherein the β-catenin degradation, which is typically induced by ubiquitin-proteosome pathway, was inhibited by Trop2/β-catenin complex. BD-mediated destabilization of β-catenin diminished nuclear translocation, which in turn decreased Trop2 transcription, reversed the epithelial-mesenchymal transition (EMT) process, and restrained the extracellular matrix (ECM) remodeling, thereby collectively impeding cancer metastasis. Furthermore, BD's efficacy in hampering lung metastatic colonization and its favorable impact on extending survival were corroborated using a 4T1 experimental metastasis model. These findings offer new insights into the discovery of anti-Trop2 inhibitors, with additional structural investigations required to confirm their detailed binding sites and MOAs.

## Trop2-targeted imaging

Trop2 overexpression is linked to a poor prognosis in a variety of solid tumors and can also predict response to different Trop2-targeted therapies 43, 48, 88. Consequently, accurate assessment of Trop2 status is critical for guiding clinical decision-making regarding treatment strategies. However, unlike other therapeutic targets, there are no standard methods for Trop2 testing when selecting patients for clinical trials, which may hinder achieving the best clinical outcomes. Below, we delve into the evolving Trop2-targeted imaging strategies that show promise for cancer diagnosis, treatment monitoring, and theranostic applications.

Sharkey and colleagues developed a novel bispecific antibody, named TF12, capable of binding to both Trop2 and histamine-succinyl-glycine (HSG) hapten 89. The moderate internalization and prolonged retention of TF12 on the surface of Trop2-expressing tumor cells rendered it suitable for pretargeted imaging and therapy in Trop2-positive tumors. As a result, Lutje *et al.* designed a pretargeted dual-modality immuno-SPECT and near-infrared fluorescence (NIRF) imaging, for image-guided surgery in prostate cancer using TF12 together with the dual-labeled di-HSG peptide (RDC018) 90. RDC018 was an IMP288 analog that contains a NIRF IRDye800CW moiety and a 1,4,7,10-tetraazacyclododecane-1,4,7,10-tetraacetic acid (DOTA) chelate, enabling radiolabeling with ^111^In. In prostate cancer metastases mouse models pre-targeted with the TF12, there was notable Trop2-specific uptake of ^111^In-RDC018, resulting in favorable signal-to-background ratios. The TF12-based pre-targeted radio-immunoimaging strategy, when combined with ^68^Ga di-HSG peptide IMP288 (^68^Ga-IMP288), achieved rapid, highly sensitive, and specific imaging in prostate cancer models 91. Van Rij and colleagues reported that the combination of TF12 and ^177^Lu-IMP288 further constituted an effective pre-targeted radio-immunotherapy (PRIT) system, which substantially prolonged survival in mouse models with PC3 xenografts 92.

Beyond pre-targeted strategy, direct targeting approaches using labeled antibodies have also exhibited promising theranostic potential for Trop2-positive solid tumors. Chen and colleagues synthesized a radiolabeled agent, ^89^Zr-DFO-AF650, by conjugating an anti-Trop2 antibody AF650 with desferrioxamine (DFO) 93. This agent demonstrated specific, rapid, and sustained accumulation in Trop2-positive tumor models, specifically BxPC-3. Notably, ^89^Zr-DFO-AF650 was effective in distinguishing primary tumors in the orthotopic BxPC-3 cancer model via immune-PET. The therapeutic antibody hIMB1636 was conjugated with p-SCN-Bn-NOTA (NOTA) and DOTA-NHS-ester (DOTA), enabling radiolabeling with ^64^Cu and ^177^Lu, respectively. *In vivo* evaluations indicated that ^64^Cu-labeled hIMB1636 could noninvasively assess Trop2 expression levels using positron emission tomography (PET) imaging, and ^177^Lu-labeled hIMB1636 significantly suppressed tumor growth in Trop2-overexpressing T3M-4 mouse models 94.

In addition to traditional mAbs, nanobodies have been used to develop tracers for Trop2-targeted molecular imaging. Huang and colleagues constructed two nanobody-based immune-PET probes (^68^Ga-NOTA-RTD98 and ^68^Ga-NOTA-RTD01) and assessed their diagnostic abilities in preclinical pancreatic and gastric cancer models. Immuno-PET with ^68^Ga-NOTA-RTD98 effectively delineated subcutaneous tumors in both cell-derived pancreatic cancer models and patient-derived gastric cancer models, demonstrating its superiority over imaging with ^18^F-FDG or the non-specific probe. Another probe, ^68^Ga-NOTA-RTD01, featuring improved pharmacokinetics targeting Trop2, was further developed and demonstrated advantageous diagnostic capabilities in preclinical pancreatic cancer models 95. Subsequently, Huang and colleagues designed an optimal ^68^Ga-labelled nanobody tracer, ^68^Ga-NOTA-T4. Preclinical studies involving tumor-bearing mouse models and beagle dogs revealed good pharmacokinetics and diagnostic potential of the tracer. Moreover, in a pilot clinical trial involving patients with solid tumors, ^68^Ga-NOTA-T4 immuno-PET noninvasively and accurately visualized varying Trop2 expression in primary and metastatic tumors 96.

These findings underscore the potential of mAb-based or nanobody-based molecular imaging in Trop2-positive solid tumors. Although mAb-based imaging offers prolonged imaging capabilities and established clinical applicability with effective effector functions, its efficiency may be compromised by poor tumor penetration, higher production costs, and potential immunogenicity. In contrast, nanobody-based imaging provides excellent tumor penetration, rapid imaging capabilities, cost-effective production, and reduced immunogenicity, though it is limited by a lack of natural effector functions and shorter systemic circulation times. For optimal imaging and treatment of Trop2-positive solid tumors, the choice between mAb-based and nanobody-based strategies likely depends on the tumor types, desired imaging windows, and required pharmacokinetics. Wu and his team recently identified a multicyclic peptide binder to Trop2 using a phage-displayed peptide library. The cysteine-framework peptide binder exhibited a nanomolar affinity for the Trop2 antigen and demonstrated specific binding to Trop2 on the cell surface 97. Given the advantages of peptides—namely their size and penetration properties, ease of synthesis and modification, versatility, customization, and lower immunogenicity—peptide-based imaging or therapeutic agents are promising for diagnosing and treating Trop2-positive tumors.

Overall, these findings highlight the role of Trop2-mediated imaging in tumor detection and monitoring of treatment responses. Advancing these Trop2-targeted imaging strategies through clinical validation and translational research could enhance the stratification and management of patients with Trop2-positive tumors.

## Conclusions and Future Directions

Trop2 has emerged as a promising target for cancer therapy due to its overexpression in cancer cells. Advances in ADC-based anti-Trop2 therapies have significantly transformed the treatment landscape for a subset of patients with Trop2-positive solid tumors. However, challenges such as systemic toxicity and drug resistance persist. The growing body of knowledge regarding Trop2-mediated oncogenic mechanisms and advances in structural biology have spurred the development of next-generation therapies. Numerous emerging Trop2-targeted modalities, such as bispecific antibodies/ADCs and CAR-T cell therapies, are undergoing active investigation in the early clinical phase, with some showing the potential to revolutionize the care of Trop2-positive cancers. In this review, we provide a summary of the components and underlying rationale of innovative Trop2-targeted therapeutics. Importantly, we examine the varied MOAs and limitations of these therapies. To fully realize the therapeutic potential of these Trop2-targeted strategies, future research endeavors ought to concentrate on pivotal areas.

**Figure 5:**
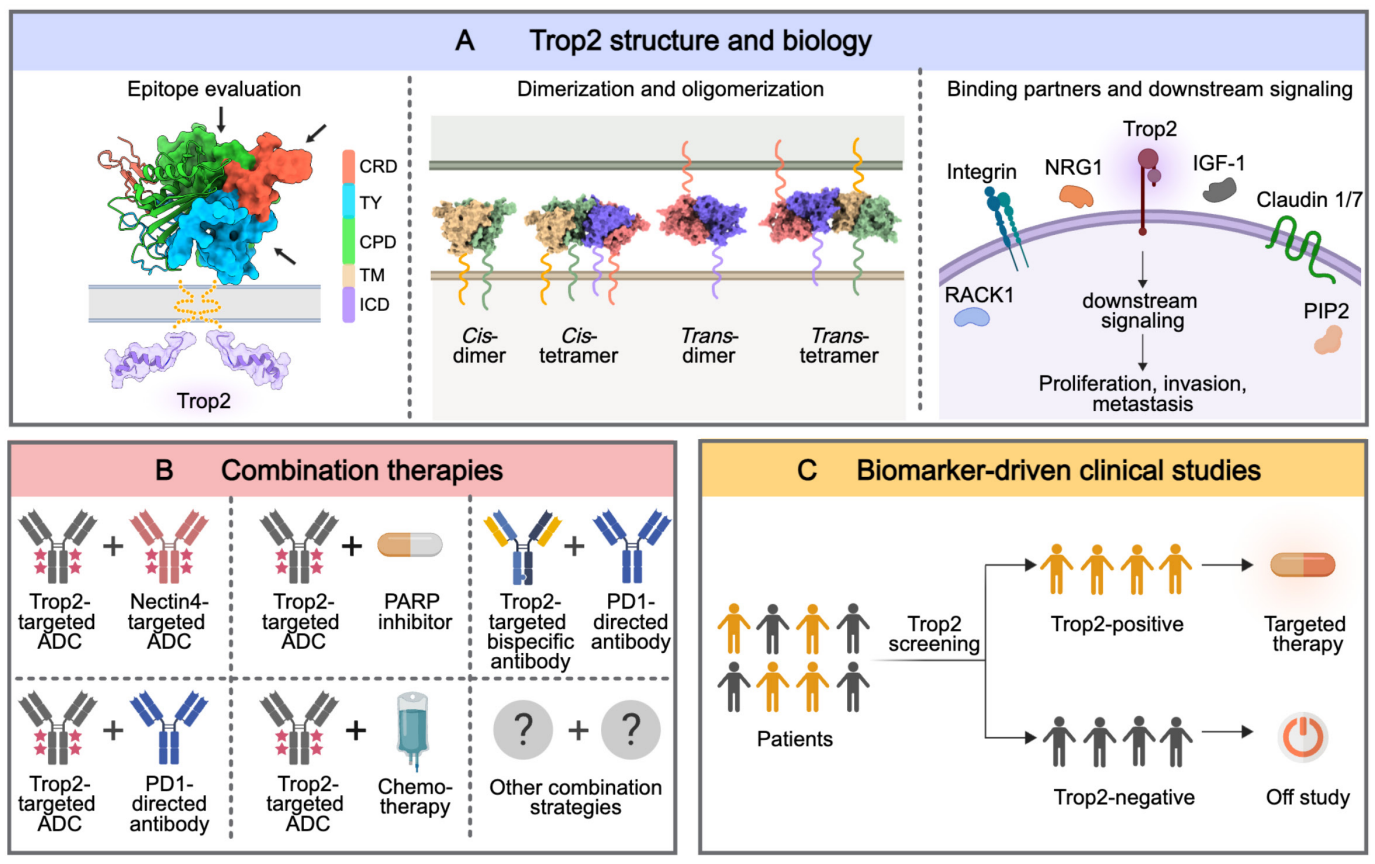
** Future directions of Trop2-targeted therapies for the treatment of Trop2-positive tumors.** (A) A deeper understanding of the structure and mechanisms of Trop2. A proposed composite model of full-length Trop2 dimer based on the structures of individual modules. Schematics for possible dimerization and tetramerization states of Trop2 on the cell surface. PDB identifier 7E5N for *cis*-dimer and *cis*-tetramer, PDB identifier 7E5M for *trans*-dimer and *trans*-tetramer. A general view of Trop2 interaction partners and downstream signaling pathways. NRG1, neuregulin 1; IGF-1, insulin-like growth factor 1; RACK1, receptor for activated C kinase 1; PIP2, phosphatidylinositol 4,5-bisphosphate. (B) Discovery of optimal combination strategies against Trop2-positive tumors. The figure shows representative examples of potential combination strategies with Trop2-targeted agents in preclinical or early clinical stages. PARP, poly (ADP-ribose) polymerase; PD1, programmed cell death protein 1. (C) Establishment of a standard method for Trop2 testing is necessary for the biomarker-driven clinical studies that can evaluate the efficacy of potential Trop2-targeted therapeutic agents. The image was created with *MedPeer.cn*.

Firstly, both basic scientists and clinicians must undertake an in-depth analysis of the oncogenic mechanisms associated with Trop2 overexpression in tumor tissues (Figure 5A). As an intricate signal transducer, Trop2 is known for its complex oligomerization and interactions with other proteins. Recent advances in structural biology, such as the crystallographic determination of Trop2's dimeric structure, have shed light on aspects of its oligomerization 45, 46. However, the relationship between Trop2's oligomeric assembly and its biological functions is not yet fully understood. The structural basis underlying interactions between Trop2 and its partners remains to be fully explained. Notably, Trop2 is not only expressed on tumor cells but also on some nonmalignant tissues, which can lead to inevitable on-target off-tumor toxicities. To enhance the tumor specificity of targeted therapies, identifying tumor-specific structural variants of Trop2 seems to be a viable way 36. In this context, concentrated efforts to evaluate Trop2 epitopes, investigate its interaction dynamics, and understand its biology would lead to the development of anti-Trop2 therapies with greater precision and clinical effectiveness.

The second consideration is exploring the potential combination strategies for therapy (Figure 5B). Owing to the heterogeneity and individual specificity of tumors, relying on a single agent or therapy is often sufficient for better outcomes. Preliminary data from preclinical and early clinical studies have shown that Trop2-direct ADCs, when combined with chemotherapeutic agents, small molecule inhibitors, immunotherapy, or anti-angiogenic therapy, can lead to synergistic antitumor effects in drug-resistant tumors 43, 88, 98. However, it should be noted that the superimposed toxicity from drug-drug interaction may elevate the risk of treatment failure. Therefore, finding the optimal combination therapies with a favorable response and manageable toxicity remains a significant challenge.

The third and final facet of future research involves establishing the standard methods of Trop2 testing, which has been overlooked for a long time during the development of Trop2-targeted therapies (Figure 5C). Indeed, there is no formal cut-off for 'Trop2 positivity'. Current Trop2-targeted therapies tend to be more effective in inhibiting tumors that exhibit medium to high levels of Trop2 expression. Therefore, patient stratification based on Trop2 status is expected to aid patient selection and further personalized medicine.

## Figures and Tables

**Table 1:** Additional Trop2-targeted ADCs in clinical development

Name	Institute/Company	Phase/Clinical trial ID	Trial design/Patient population	Reference
BIO-106	BiOneCure Therapeutics	Phase 1/2/ NCT05320588	BIO-106 or BIO-106 + pembrolizumab in advanced cancers	Not applicable
BL-M02D1	Biokin Pharmaceutical	Phase 1/2/ NCT05339685, NCT05385692, NCT05949619	BL-M02D1 in locally advanced or metastatic TNBC or other solid tumors (NCT05339685); BL-M02D1 in locally advanced or metastatic gastroenteric tumors or other solid tumors (NCT05385692); BL-M02D1 in locally advanced or metastatic NSCLC or other solid tumors (NCT05949619)	99
9MW2921	Mabwell (Shanghai) Bioscience	Phase 1/2/ NCT05990452	9MW2921 in locally advanced or metastatic solid tumors refractory to all standard therapies	Not applicable
DB-1305	Yinen Biopharmaceutical	Phase 1/2/ NCT05438329	DB-1305 in advanced/unresectable, recurrent, or metastatic malignant solid tumors	100
IBI130	Innovent Biologics	Phase 1/2/ NCT05923008	IBI130 in unresectable, locally advanced, or metastatic solid tumors	Not applicable
HS-20105	Hansoh Pharma	Phase 1/ NCT06144723	HS-20105 in advanced solid tumors	Not applicable
BAT8008	Bio-Thera Solutions	Phase 1/ NCT05620017	BAT8008 in advanced solid tumors	101
FDA018	Shanghai Fudan Zhangjiang Bio Pharmaceutical	Phase 1/ NCT05174637	FDA018 in advanced solid tumors	Not applicable
FZ-AD004	Shanghai Fudan-Zhangjiang Bio-Pharmaceutical	Phase 1/ NCT05914545	FZ-AD004 in advanced/metastatic solid tumors	Not applicable
JS108	Hangzhou DAC Biotechnology	Phase 1/ NCT04601285	JS108 in advanced solid tumors	Not applicable
BAT8003	Bio-Thera Solutions	Phase 1/ NCT03884517, terminated	BAT8003 in advanced epithelial cancer	102

TNBC, triple-negative breast cancer; NSCLC, non-small cell lung cancer.

**Table 2:** Additional Trop2-targeted monoclonal antibodies in preclinical development

Name	Institute/ Company	Reference/ Patent
OXG-64	Oncoxx Biotech SRL	WO201089782A1
KD065	Nanjing KAEDI Biotech	Not applicable
Harbour Biomed patent anti-Trop2 antibody	Harbour Biomed	WO2022143670
Kisoji Bio patent anti-Trop2 antibody	Kisoji Biotechnology	WO2023049999
WuXi Biologics patent anti-Trop2 antibody	WuXi Biologics (Shanghai)	WO2022152308
Chia Tai Tianqing Pharma patent anti-Trop2 antibody	Chia Tai Tianqing Pharmaceutical Group	WO2023104080
Biosion patent anti-Trop2 antibody	Biosion	WO2022222992
Zhejiang Nanomab Tech. patent anti-Trop2-antibody	Zhejiang Nanomab Technology Center	WO2023274365

**Table 3:** Bispecific or trispecific agents in preclinical development for Trop2-expressing tumors

Name	Institute/ Company	Type	Target	Reference/ Patent
(E1)-3s	Immunomedics	Bispecific antibody	Trop2, CD3	69, 70
F7AK3	Huazhong University of Science and Technology Tongji Medical College	Bispecific antibody	Trop2, CD3	71
Trop2/CD3 bispecific antibody	Arbele Corp	Bispecific antibody	Trop2, CD3	73
Sunshine Guojian anti-Trop2/CD3 bispecific antibody	Sunshine Guojian Pharmaceutical (Shanghai)	Bispecific antibody	Trop2, CD3	72
EX108	Excelmab	Bispecific antibody	Trop2, CD3	WO2019184549A1
Janux patent anti-Trop2/CD3 bispecific antibody	Janux Therapeutics	Bispecific antibody	Trop2, CD3	WO2023164513
BiOneCure patent anti-Trop2/HER2 bispecific antibody	BiOneCure Therapeutics	Bispecific antibody	Trop2, HER2	WO2022159984
Zeda Bio patent anti-Trop2/PD-L1 bispecific antibody	Zeda Biopharmaceuticals	Bispecific antibody	Trop2, PD-L1	WO2023098770
Xencor patent anti-Trop2/CD28 bispecific antibody	Xencor	Bispecific antibody	Trop2, CD28	WO2023164640
TF12	Radboud University Medical Center	Bispecific antibody	Trop2, HSG	90
Trop2 ProTriTAC	Harpoon Therapeutics	Trispecific antibody	Trop2, CD3, HSA	74
Boehringer patent anti-Trop2/CD3/CDH-17 trispecific antibody	Boehringer Ingelheim	Trispecific antibody	Trop2, CD3, CDH-17	WO2022263507
YH012	Beijing Biocytogen	Bispecific ADC	Trop2, HER2	76
BCG033	Beijing Biocytogen	Bispecific ADC	Trop2, PTK7	79
BCG011	Beijing Biocytogen	Bispecific ADC	Trop2, EGFR	WO2023046061A1
DM001	Xadcera Biopharmaceutical	Bispecific ADC	Trop2, EGFR	75
JSKN016	Alphamab Oncology	Bispecific ADC	Trop2, HER3	Not applicable
HuNb_TROP2-HSA_-MMAE	Shanghai Novamab Biopharmaceuticals	Bispecific ADC	Trop2, HSA	80

HER2, human epidermal growth factor receptor 2; PD-L1, programmed death-ligand 1; HSG, histamine-succinyl-glycine; CDH-17, cadherin-17; PTK7, protein tyrosine kinase-7; EGFR, epidermal growth factor receptor; HER3, human epidermal growth factor receptor 3;HSA human serum albumin.

**Table 4:** CAR-T and CAR-NK therapies in preclinical and early clinical stage

Name	Institute/ Company	Type	Phase/ clinical trial ID	Reference/ Patent
T60c	Cancer Hospital Chinese Academy of Medical Sciences	CAR-T	Phase 1/2/ NCT06082557	Not applicable
MT-302	Myeloid Therapeutics	CAR-T	Phase 1/ NCT05969041	Not applicable
Immunomedics anti-Trop-2 CAR	Immunomedics	CAR-T	Preclinical	US20160361360
Trop2/PD-L1 CAR-T	Nanjing Medical University	CAR-T	Preclinical	81
Trop2-CAR-NK	M.D. Anderson Cancer Center	CAR-NK	Phase 1/2/ NCT05922930	Not applicable
Trop2-CAR	German Cancer Research Center	CAR-NK	Preclinical	82

NK, natural killer; CAR, chimeric antigen receptor.

## Abbreviations

Trop2: trophoblast cell surface antigen 2
ADC: antibody-drug conjugate
TNBC: triple-negative breast cancer
MOA: mechanism of action
EGP-1: epithelial glycoprotein-1
M1S1: membrane component chromosome 1 surface marker 1
mBC: metastatic breast cancer
mUC: metastatic urothelial cancer
SG: Sacituzumab govitecan
FIC: first-in-class
DAR: drug-to-antibody ratio
FDA: Food and Drug Administration
HER2: human epidermal growth factor receptor 2
HR: hormone receptor
ORR: objective response rate
DAD: Double Antibody Drug Conjugate
EV: enfortumab vedotin
AEs: SG-related adverse events
OS: overall survival
NSCLC: non-small cell lung cancer
mAb: monoclonal antibody
PDX: patient-derived xenograft
BC: breast cancer
FIH: first-in-human
PFS: progression-free survival
cORR: confirmed ORR
AGAs: actionable genomic alterations
ILD: interstitial lung disease
IRRs: infusion-related reactions
OSEs: ocular surface events
CDX: cell-derived xenograft
PK-PD: pharmacokinetic-pharmacodynamic
DoR: duration of response
TKI: tyrosine kinase inhibitor
EGFR: epidermal growth factor receptor
DCR: disease control rate
DLT: dose-limiting toxicity
MMAE: monomethyl auristatin E
PDAC: pancreatic ductal adenocarcinoma
TI: therapeutic index
PD-1: programmed cell death 1
TRAEs: treatment-related adverse events
ECD: extracellular domain
CPD: cysteine-poor domain
ADAM10: ADAM metallopeptidase domain 10
MAPK: mitogen-activated protein kinase
CDC: complement-dependent cytotoxicity
CRD: cysteine-rich domain
ADCC: antibody-dependent cellular cytotoxicity
CLIPS: chemically linked peptides on scaffolds
CBIS: Cell‑Based Immunization and Screening
Nb: nanobody
TY: thyroglobulin type-1
NHP: non-human primate
BiTEs: bispecific T cell engagers
INFα: interferon-α
TME: tumor microenvironment
CDR: complementarity-determining region
CDH-17: cadherin-17
GC: gastric cancer
HER3: human epidermal growth factor receptor 3
PI3K: phosphatidylinositide-3 kinase
CDE: China's Center for Drug Evaluation
PTK7: Protein tyrosine kinase-7
HSA: human serum albumin
CARs: chimeric antigen receptors
PBMCs: peripheral blood mononuclear cells
NK cells: natural killer cells
VLPs: virus-like particles
SIV: simian immunodeficiency virus
NKT cells: natural killer T cells
TILs: tumor-infiltrating lymphocytes
Treg cells: regulatory T cells
MDSCs: myeloid-derived suppressor cells
IL-10: Interleukin 10
TGF-β: transforming growth factor-β
HIV: human immunodeficiency virus
DCs: dendritic cells
ODEX: oxidized dextran
BD: Bruceine D
CMC: cell membrane chromatography
CETSA: cellular thermal shift assay
ICD: intracellular domain
EMT: epithelial-mesenchymal transition
ECM: extracellular matrix
HSG: histamine-succinyl-glycine
NIRF: near-infrared fluorescence
DOTA: 1,4,7,10-tetraazacyclododecane-1,4,7,10-tetraacetic acid
PRIT: pretargeted radio-immunotherapy
DFO: desferrioxamine
PET: positron emission tomography
